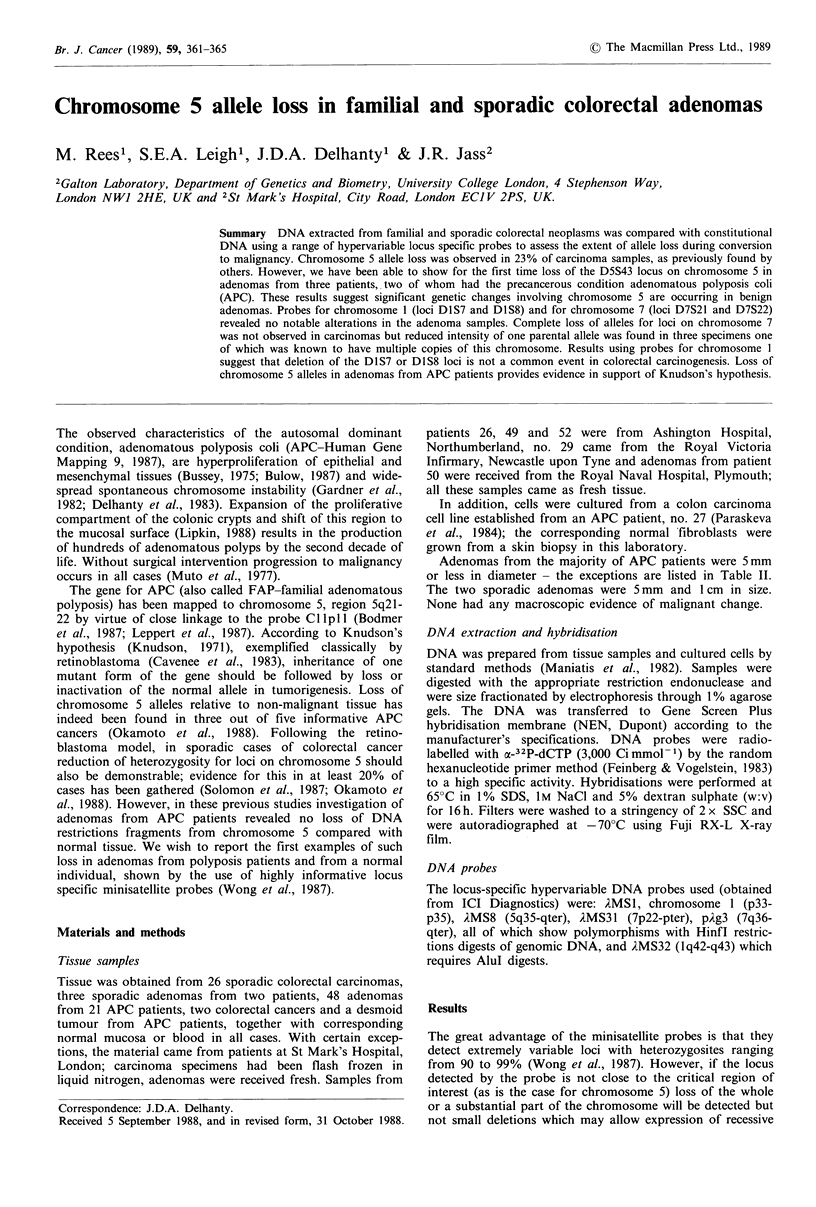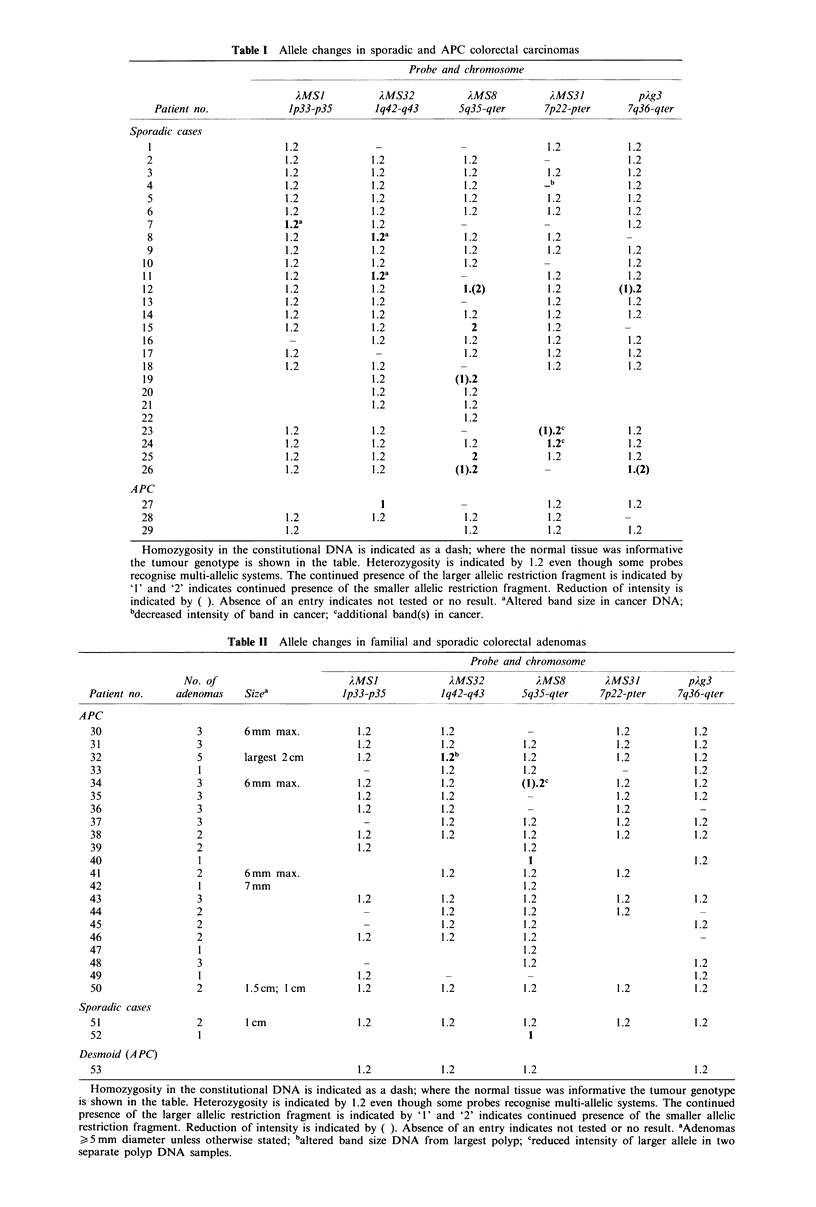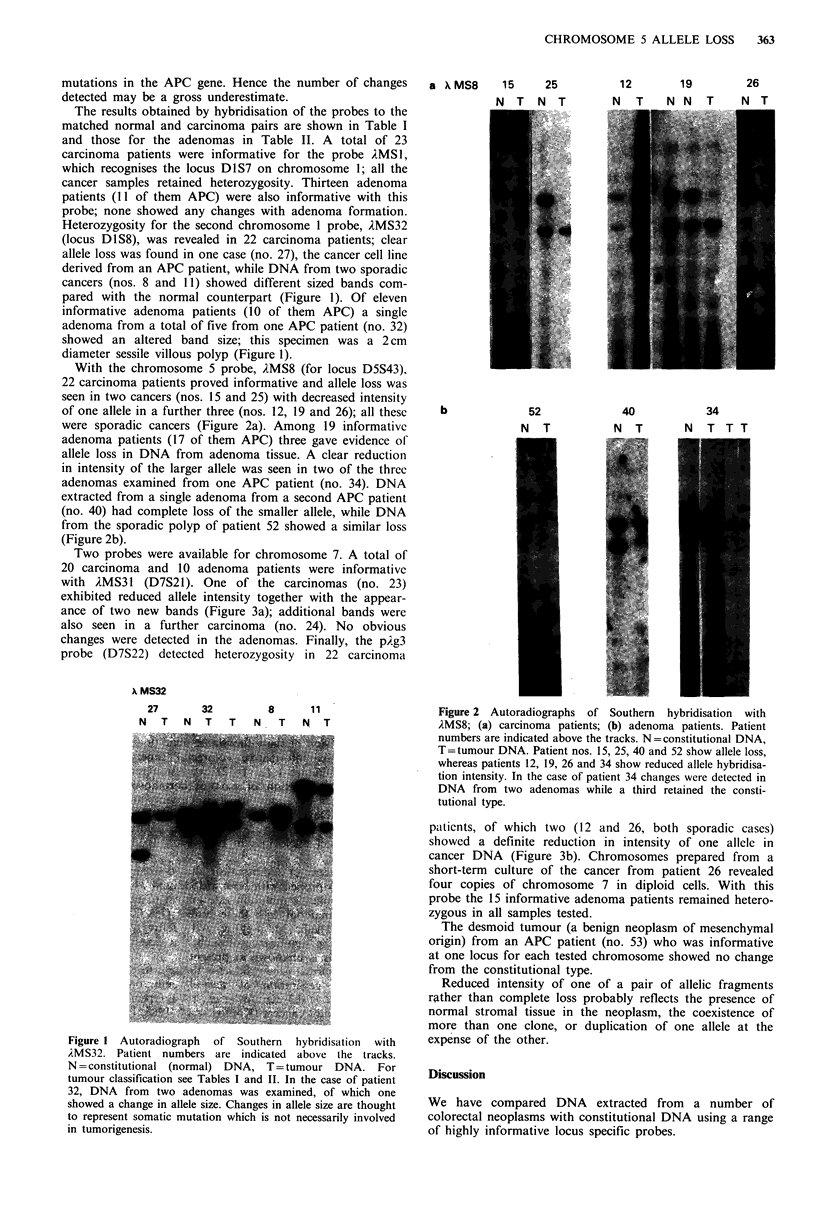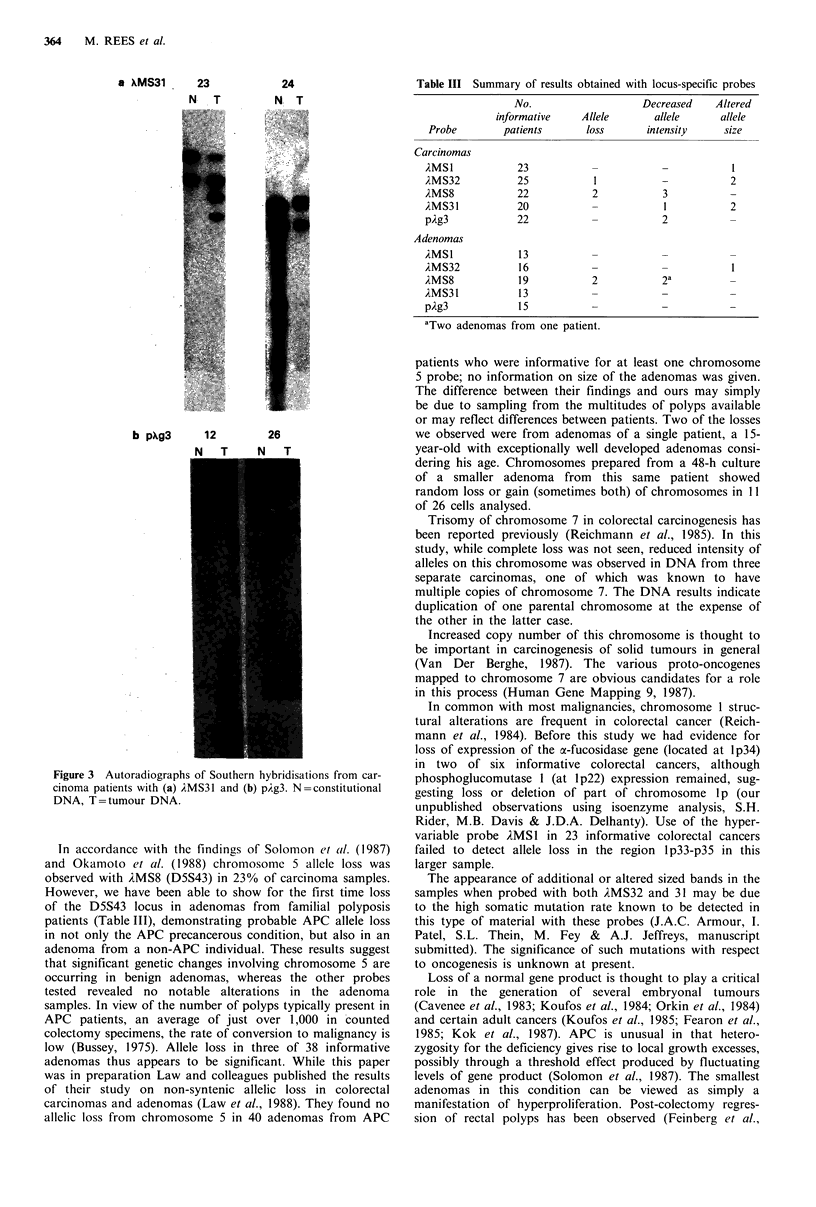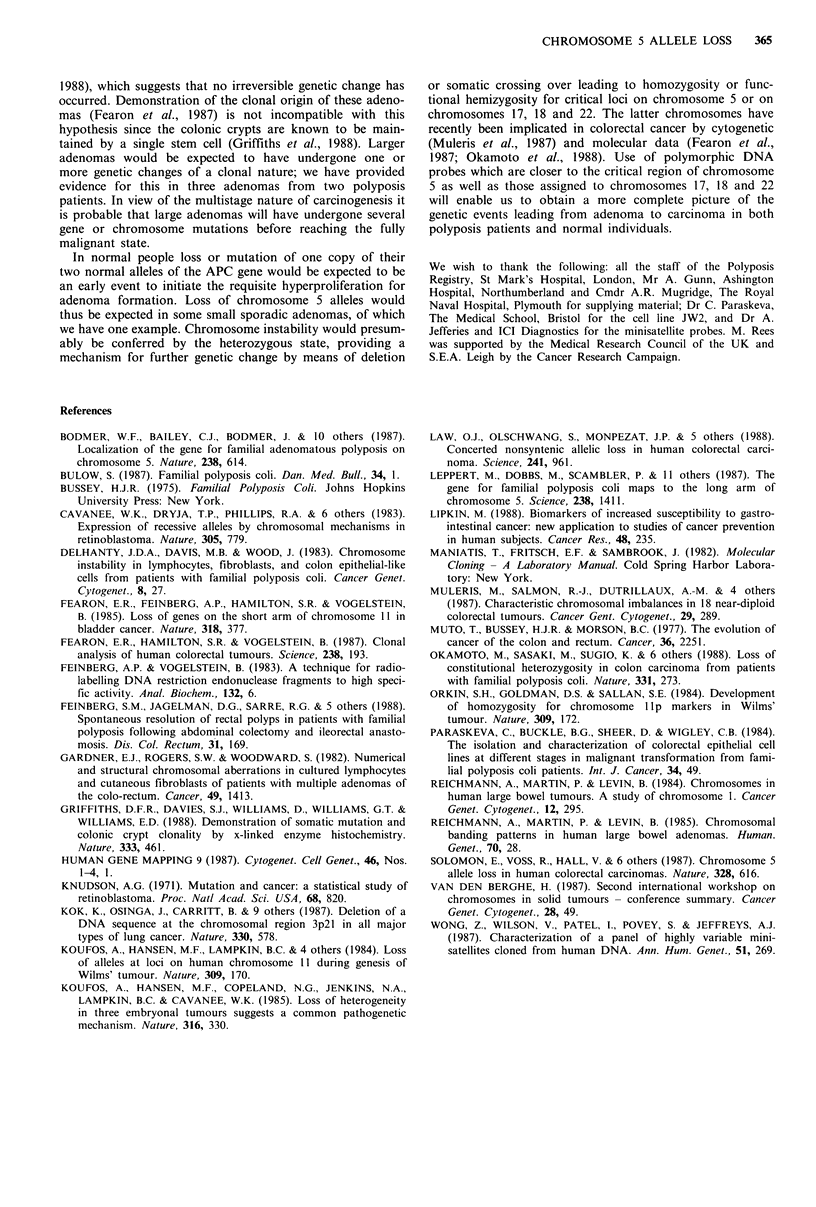# Chromosome 5 allele loss in familial and sporadic colorectal adenomas.

**DOI:** 10.1038/bjc.1989.72

**Published:** 1989-03

**Authors:** M. Rees, S. E. Leigh, J. D. Delhanty, J. R. Jass

**Affiliations:** Galton Laboratory, Department of Genetics and Biometry, University College London, UK.

## Abstract

**Images:**


Br. J. Cancer (1989), 59, 361–365 © The Macmillan Press Ltd., 1989

### Chromosome 5 allele loss in familial and sporadic colorectal adenomas

M. Rees[1], S.E.A. Leigh[1], J.D.A. Delhanty[1] & J.R. Jass[2]

[2]Galton Laboratory, Department of Genetics and Biometry, University College London, 4 Stephenson Way, London NW1 2HE, UK and [2]St Mark's Hospital, City Road, London EC1V 2PS, UK.

**Summary** DNA extracted from familial and sporadic colorectal neoplasms was compared with constitutional DNA using a range of hypervariable locus specific probes to assess the extent of allele loss during conversion to malignancy. Chromosome 5 allele loss was observed in 23% of carcinoma samples, as previously found by others. However, we have been able to show for the first time loss of the D5S43 locus on chromosome 5 in adenomas from three patients, two of whom had the precancerous condition adenomatous polyposis coli (APC). These results suggest significant genetic changes involving chromosome 5 are occurring in benign adenomas. Probes for chromosome 1 (loci D1S7 and D1S8) and for chromosome 7 (loci D7S21 and D7S22) revealed no notable alterations in the adenoma samples. Complete loss of alleles for loci on chromosome 7 was not observed in carcinomas but reduced intensity of one parental allele was found in three specimens one of which was known to have multiple copies of this chromosome. Results using probes for chromosome 1 suggest that deletion of the D1S7 or D1S8 loci is not a common event in colorectal carcinogenesis. Loss of chromosome 5 alleles in adenomas from APC patients provides evidence in support of Knudson's hypothesis.

The observed characteristics of the autosomal dominant condition, adenomatous polyposis coli (APC–Human Gene Mapping 9, 1987), are hyperproliferation of epithelial and mesenchymal tissues (Bussey, 1975; Bulow, 1987) and widespread spontaneous chromosome instability (Gardner *et al.*, 1982; Delhanty *et al.*, 1983). Expansion of the proliferative compartment of the colonic crypts and shift of this region to the mucosal surface (Lipkin, 1988) results in the production of hundreds of adenomatous polyps by the second decade of life. Without surgical intervention progression to malignancy occurs in all cases (Muto *et al.*, 1977).

The gene for APC (also called FAP–familial adenomatous polyposis) has been mapped to chromosome 5, region 5q21-22 by virtue of close linkage to the probe C11p11 (Bodmer *et al.*, 1987; Leppert *et al.*, 1987). According to Knudson's hypothesis (Knudson, 1971), exemplified classically by retinoblastoma (Cavenee *et al.*, 1983), inheritance of one mutant form of the gene should be followed by loss or inactivation of the normal allele in tumorigenesis. Loss of chromosome 5 alleles relative to non-malignant tissue has indeed been found in three out of five informative APC cancers (Okamoto *et al.*, 1988). Following the retinoblastoma model, in sporadic cases of colorectal cancer reduction of heterozygosity for loci on chromosome 5 should also be demonstrable; evidence for this in at least 20% of cases has been gathered (Solomon *et al.*, 1987; Okamoto *et al.*, 1988). However, in these previous studies investigation of adenomas from APC patients revealed no loss of DNA restrictions fragments from chromosome 5 compared with normal tissue. We wish to report the first examples of such loss in adenomas from polyposis patients and from a normal individual, shown by the use of highly informative locus specific minisatellite probes (Wong *et al.*, 1987).

### Materials and methods

### *Tissue samples*

Tissue was obtained from 26 sporadic colorectal carcinomas, three sporadic adenomas from two patients, 48 adenomas from 21 APC patients, two colorectal cancers and a desmoid tumour from APC patients, together with corresponding normal mucosa or blood in all cases. With certain exceptions, the material came from patients at St Mark's Hospital, London; carcinoma specimens had been flash frozen in liquid nitrogen, adenomas were received fresh. Samples from patients 26, 49 and 52 were from Ashington Hospital, Northumberland, no. 29 came from the Royal Victoria Infirmary, Newcastle upon Tyne and adenomas from patient 50 were received from the Royal Naval Hospital, Plymouth; all these samples came as fresh tissue.

In addition, cells were cultured from a colon carcinoma cell line established from an APC patient, no. 27 (Paraskeva *et al.*, 1984); the corresponding normal fibroblasts were grown from a skin biopsy in this laboratory.

Adenomas from the majority of APC patients were 5 mm or less in diameter – the exceptions are listed in Table II. The two sporadic adenomas were 5 mm and 1 cm in size. None had any macroscopic evidence of malignant change.

### *DNA extraction and hybridisation*

DNA was prepared from tissue samples and cultured cells by standard methods (Maniatis *et al.*, 1982). Samples were digested with the appropriate restriction endonuclease and were size fractionated by electrophoresis through 1% agarose gels. The DNA was transferred to Gene Screen Plus hybridisation membrane (NEN, Dupont) according to the manufacturer's specifications. DNA probes were radiolabelled with $\alpha$-$^{32}$P-dCTP (3,000 Ci mmol$^{-1}$) by the random hexanucleotide primer method (Feinberg & Vogelstein, 1983) to a high specific activity. Hybridisations were performed at 65°C in 1% SDS, 1 M NaCl and 5% dextran sulphate (w:v) for 16 h. Filters were washed to a stringency of 2× SSC and were autoradiographed at −70°C using Fuji RX-L X-ray film.

### *DNA probes*

The locus-specific hypervariable DNA probes used (obtained from ICI Diagnostics) were: λMS1, chromosome 1 (p33-p35), λMS8 (5q35-qter), λMS31 (7p22-pter), pλg3 (7q36-qter), all of which show polymorphisms with HinfI restrictions digests of genomic DNA, and λMS32 (1q42-q43) which requires AluI digests.

### Results

The great advantage of the minisatellite probes is that they detect extremely variable loci with heterozygosites ranging from 90 to 99% (Wong *et al.*, 1987). However, if the locus detected by the probe is not close to the critical region of interest (as is the case for chromosome 5) loss of the whole or a substantial part of the chromosome will be detected but not small deletions which may allow expression of recessive

Correspondence: J.D.A. Delhanty.
Received 5 September 1988, and in revised form, 31 October 1988.

**Table I** Allele changes in sporadic and APC colorectal carcinomas

| Patient no. | *Probe and chromosome* | | | | |
|---|---|---|---|---|---|
| | *λMS1* *1p33-p35* | *λMS32* *1q42-q43* | *λMS8* *5q35-qter* | *λMS31* *7p22-pter* | *pλg3* *7q36-qter* |
| *Sporadic cases* | | | | | |
| 1 | 1.2 | – | – | 1.2 | 1.2 |
| 2 | 1.2 | 1.2 | 1.2 | – | 1.2 |
| 3 | 1.2 | 1.2 | 1.2 | 1.2 | 1.2 |
| 4 | 1.2 | 1.2 | 1.2 | –[b] | 1.2 |
| 5 | 1.2 | 1.2 | 1.2 | 1.2 | 1.2 |
| 6 | 1.2 | 1.2 | 1.2 | 1.2 | 1.2 |
| 7 | **1.2**[a] | 1.2 | – | – | 1.2 |
| 8 | 1.2 | **1.2**[a] | 1.2 | 1.2 | – |
| 9 | 1.2 | 1.2 | 1.2 | 1.2 | 1.2 |
| 10 | 1.2 | 1.2 | 1.2 | – | 1.2 |
| 11 | 1.2 | **1.2**[a] | – | 1.2 | 1.2 |
| 12 | 1.2 | 1.2 | **1.(2)** | 1.2 | **(1).2** |
| 13 | 1.2 | 1.2 | – | 1.2 | 1.2 |
| 14 | 1.2 | 1.2 | 1.2 | 1.2 | 1.2 |
| 15 | 1.2 | 1.2 | **2** | 1.2 | – |
| 16 | – | 1.2 | 1.2 | 1.2 | 1.2 |
| 17 | 1.2 | – | 1.2 | 1.2 | 1.2 |
| 18 | 1.2 | 1.2 | – | 1.2 | 1.2 |
| 19 | | 1.2 | **(1).2** | | |
| 20 | | 1.2 | 1.2 | | |
| 21 | | 1.2 | 1.2 | | |
| 22 | | | 1.2 | | |
| 23 | 1.2 | 1.2 | – | **(1).2**[c] | 1.2 |
| 24 | 1.2 | 1.2 | 1.2 | **1.2**[c] | 1.2 |
| 25 | 1.2 | 1.2 | **2** | 1.2 | 1.2 |
| 26 | 1.2 | 1.2 | **(1).2** | – | **1.(2)** |
| *APC* | | | | | |
| 27 | | **1** | – | 1.2 | 1.2 |
| 28 | 1.2 | 1.2 | 1.2 | 1.2 | – |
| 29 | 1.2 | | 1.2 | 1.2 | 1.2 |

Homozygosity in the constitutional DNA is indicated as a dash; where the normal tissue was informative the tumour genotype is shown in the table. Heterozygosity is indicated by 1.2 even though some probes recognise multi-allelic systems. The continued presence of the larger allelic restriction fragment is indicated by '1' and '2' indicates continued presence of the smaller allelic restriction fragment. Reduction of intensity is indicated by ( ). Absence of an entry indicates not tested or no result. [a]Altered band size in cancer DNA; [b]decreased intensity of band in cancer; [c]additional band(s) in cancer.

**Table II** Allele changes in familial and sporadic colorectal adenomas

| Patient no. | No. of adenomas | Size[a] | *Probe and chromosome* | | | | |
|---|---|---|---|---|---|---|---|
| | | | *λMS1* *1p33-p35* | *λMS32* *1q42-q43* | *λMS8* *5q35-qter* | *λMS31* *7p22-pter* | *pλg3* *7q36-qter* |
| *APC* | | | | | | | |
| 30 | 3 | 6 mm max. | 1.2 | 1.2 | – | 1.2 | 1.2 |
| 31 | 3 | | 1.2 | 1.2 | 1.2 | 1.2 | 1.2 |
| 32 | 5 | largest 2 cm | 1.2 | **1.2**[b] | 1.2 | 1.2 | 1.2 |
| 33 | 1 | | – | 1.2 | 1.2 | – | 1.2 |
| 34 | 3 | 6 mm max. | 1.2 | 1.2 | **(1).2**[c] | 1.2 | 1.2 |
| 35 | 3 | | 1.2 | 1.2 | – | 1.2 | 1.2 |
| 36 | 3 | | 1.2 | 1.2 | – | 1.2 | – |
| 37 | 3 | | – | 1.2 | 1.2 | 1.2 | 1.2 |
| 38 | 2 | | 1.2 | 1.2 | 1.2 | 1.2 | 1.2 |
| 39 | 2 | | 1.2 | | 1.2 | | |
| 40 | 1 | | | | **1** | | 1.2 |
| 41 | 2 | 6 mm max. | | 1.2 | 1.2 | 1.2 | |
| 42 | 1 | 7 mm | | | 1.2 | | |
| 43 | 3 | | 1.2 | 1.2 | 1.2 | 1.2 | 1.2 |
| 44 | 2 | | – | 1.2 | 1.2 | 1.2 | – |
| 45 | 2 | | – | 1.2 | 1.2 | | 1.2 |
| 46 | 2 | | 1.2 | 1.2 | 1.2 | | – |
| 47 | 1 | | | | 1.2 | | |
| 48 | 3 | | – | | 1.2 | | 1.2 |
| 49 | 1 | | 1.2 | – | – | | 1.2 |
| 50 | 2 | 1.5 cm; 1 cm | 1.2 | 1.2 | 1.2 | 1.2 | 1.2 |
| *Sporadic cases* | | | | | | | |
| 51 | 2 | 1 cm | 1.2 | 1.2 | 1.2 | 1.2 | 1.2 |
| 52 | 1 | | | | **1** | | |
| *Desmoid (APC)* | | | | | | | |
| 53 | | | 1.2 | 1.2 | 1.2 | | 1.2 |

Homozygosity in the constitutional DNA is indicated as a dash; where the normal tissue was informative the tumour genotype is shown in the table. Heterozygosity is indicated by 1.2 even though some probes recognise multi-allelic systems. The continued presence of the larger allelic restriction fragment is indicated by '1' and '2' indicates continued presence of the smaller allelic restriction fragment. Reduction of intensity is indicated by ( ). Absence of an entry indicates not tested or no result. [a]Adenomas ⩾5 mm diameter unless otherwise stated; [b]altered band size DNA from largest polyp; [c]reduced intensity of larger allele in two separate polyp DNA samples.

mutations in the APC gene. Hence the number of changes detected may be a gross underestimate.

The results obtained by hybridisation of the probes to the matched normal and carcinoma pairs are shown in Table I and those for the adenomas in Table II. A total of 23 carcinoma patients were informative for the probe λMS1, which recognises the locus D1S7 on chromosome 1; all the cancer samples retained heterozygosity. Thirteen adenoma patients (11 of them APC) were also informative with this probe; none showed any changes with adenoma formation. Heterozygosity for the second chromosome 1 probe, λMS32 (locus D1S8), was revealed in 22 carcinoma patients; clear allele loss was found in one case (no. 27), the cancer cell line derived from an APC patient, while DNA from two sporadic cancers (nos. 8 and 11) showed different sized bands compared with the normal counterpart (Figure 1). Of eleven informative adenoma patients (10 of them APC) a single adenoma from a total of five from one APC patient (no. 32) showed an altered band size; this specimen was a 2 cm diameter sessile villous polyp (Figure 1).

With the chromosome 5 probe, λMS8 (for locus D5S43), 22 carcinoma patients proved informative and allele loss was seen in two cancers (nos. 15 and 25) with decreased intensity of one allele in a further three (nos. 12, 19 and 26); all these were sporadic cancers (Figure 2a). Among 19 informative adenoma patients (17 of them APC) three gave evidence of allele loss in DNA from adenoma tissue. A clear reduction in intensity of the larger allele was seen in two of the three adenomas examined from one APC patient (no. 34). DNA extracted from a single adenoma from a second APC patient (no. 40) had complete loss of the smaller allele, while DNA from the sporadic polyp of patient 52 showed a similar loss (Figure 2b).

Two probes were available for chromosome 7. A total of 20 carcinoma and 10 adenoma patients were informative with λMS31 (D7S21). One of the carcinomas (no. 23) exhibited reduced allele intensity together with the appearance of two new bands (Figure 3a); additional bands were also seen in a further carcinoma (no. 24). No obvious changes were detected in the adenomas. Finally, the pλg3 probe (D7S22) detected heterozygosity in 22 carcinoma patients, of which two (12 and 26, both sporadic cases) showed a definite reduction in intensity of one allele in cancer DNA (Figure 3b). Chromosomes prepared from a short-term culture of the cancer from patient 26 revealed four copies of chromosome 7 in diploid cells. With this probe the 15 informative adenoma patients remained heterozygous in all samples tested.

The desmoid tumour (a benign neoplasm of mesenchymal origin) from an APC patient (no. 53) who was informative at one locus for each tested chromosome showed no change from the constitutional type.

Reduced intensity of one of a pair of allelic fragments rather than complete loss probably reflects the presence of normal stromal tissue in the neoplasm, the coexistence of more than one clone, or duplication of one allele at the expense of the other.

**Figure 2** Autoradiographs of Southern hybridisation with λMS8; (**a**) carcinoma patients; (**b**) adenoma patients. Patient numbers are indicated above the tracks. N = constitutional DNA, T = tumour DNA. Patient nos. 15, 25, 40 and 52 show allele loss, whereas patients 12, 19, 26 and 34 show reduced allele hybridisation intensity. In the case of patient 34 changes were detected in DNA from two adenomas while a third retained the constitutional type.

**Figure 1** Autoradiograph of Southern hybridisation with λMS32. Patient numbers are indicated above the tracks. N = constitutional (normal) DNA, T = tumour DNA. For tumour classification see Tables I and II. In the case of patient 32, DNA from two adenomas was examined, of which one showed a change in allele size. Changes in allele size are thought to represent somatic mutation which is not necessarily involved in tumorigenesis.

### Discussion

We have compared DNA extracted from a number of colorectal neoplasms with constitutional DNA using a range of highly informative locus specific probes.

**Figure 3** Autoradiographs of Southern hybridisations from carcinoma patients with (**a**) λMS31 and (**b**) pλg3. N = constitutional DNA, T = tumour DNA.

In accordance with the findings of Solomon *et al.* (1987) and Okamoto *et al.* (1988) chromosome 5 allele loss was observed with λMS8 (D5S43) in 23% of carcinoma samples. However, we have been able to show for the first time loss of the D5S43 locus in adenomas from familial polyposis patients (Table III), demonstrating probable APC allele loss in not only the APC precancerous condition, but also in an adenoma from a non-APC individual. These results suggest that significant genetic changes involving chromosome 5 are occurring in benign adenomas, whereas the other probes tested revealed no notable alterations in the adenoma samples. In view of the number of polyps typically present in APC patients, an average of just over 1,000 in counted colectomy specimens, the rate of conversion to malignancy is low (Bussey, 1975). Allele loss in three of 38 informative adenomas thus appears to be significant. While this paper was in preparation Law and colleagues published the results of their study on non-syntenic allelic loss in colorectal carcinomas and adenomas (Law *et al.*, 1988). They found no allelic loss from chromosome 5 in 40 adenomas from APC

**Table III** Summary of results obtained with locus-specific probes

| *Probe* | *No. informative patients* | *Allele loss* | *Decreased allele intensity* | *Altered allele size* |
|---|---|---|---|---|
| *Carcinomas* | | | | |
| λMS1 | 23 | – | – | 1 |
| λMS32 | 25 | 1 | – | 2 |
| λMS8 | 22 | 2 | 3 | – |
| λMS31 | 20 | – | 1 | 2 |
| pλg3 | 22 | – | 2 | – |
| *Adenomas* | | | | |
| λMS1 | 13 | – | – | – |
| λMS32 | 16 | – | – | 1 |
| λMS8 | 19 | 2 | 2[a] | – |
| λMS31 | 13 | – | – | – |
| pλg3 | 15 | – | – | – |

[a]Two adenomas from one patient.

patients who were informative for at least one chromosome 5 probe; no information on size of the adenomas was given. The difference between their findings and ours may simply be due to sampling from the multitudes of polyps available or may reflect differences between patients. Two of the losses we observed were from adenomas of a single patient, a 15-year-old with exceptionally well developed adenomas considering his age. Chromosomes prepared from a 48-h culture of a smaller adenoma from this same patient showed random loss or gain (sometimes both) of chromosomes in 11 of 26 cells analysed.

Trisomy of chromosome 7 in colorectal carcinogenesis has been reported previously (Reichmann *et al.*, 1985). In this study, while complete loss was not seen, reduced intensity of alleles on this chromosome was observed in DNA from three separate carcinomas, one of which was known to have multiple copies of chromosome 7. The DNA results indicate duplication of one parental chromosome at the expense of the other in the latter case.

Increased copy number of this chromosome is thought to be important in carcinogenesis of solid tumours in general (Van Der Berghe, 1987). The various proto-oncogenes mapped to chromosome 7 are obvious candidates for a role in this process (Human Gene Mapping 9, 1987).

In common with most malignancies, chromosome 1 structural alterations are frequent in colorectal cancer (Reichmann *et al.*, 1984). Before this study we had evidence for loss of expression of the α-fucosidase gene (located at 1p34) in two of six informative colorectal cancers, although phosphoglucomutase 1 (at 1p22) expression remained, suggesting loss or deletion of part of chromosome 1p (our unpublished observations using isoenzyme analysis, S.H. Rider, M.B. Davis & J.D.A. Delhanty). Use of the hypervariable probe λMS1 in 23 informative colorectal cancers failed to detect allele loss in the region 1p33-p35 in this larger sample.

The appearance of additional or altered sized bands in the samples when probed with both λMS32 and 31 may be due to the high somatic mutation rate known to be detected in this type of material with these probes (J.A.C. Armour, I. Patel, S.L. Thein, M. Fey & A.J. Jeffreys, manuscript submitted). The significance of such mutations with respect to oncogenesis is unknown at present.

Loss of a normal gene product is thought to play a critical role in the generation of several embryonal tumours (Cavenee *et al.*, 1983; Koufos *et al.*, 1984; Orkin *et al.*, 1984) and certain adult cancers (Koufos *et al.*, 1985; Fearon *et al.*, 1985; Kok *et al.*, 1987). APC is unusual in that heterozygosity for the deficiency gives rise to local growth excesses, possibly through a threshold effect produced by fluctuating levels of gene product (Solomon *et al.*, 1987). The smallest adenomas in this condition can be viewed as simply a manifestation of hyperproliferation. Post-colectomy regression of rectal polyps has been observed (Feinberg *et al.*,

1988), which suggests that no irreversible genetic change has occurred. Demonstration of the clonal origin of these adenomas (Fearon *et al.*, 1987) is not incompatible with this hypothesis since the colonic crypts are known to be maintained by a single stem cell (Griffiths *et al.*, 1988). Larger adenomas would be expected to have undergone one or more genetic changes of a clonal nature; we have provided evidence for this in three adenomas from two polyposis patients. In view of the multistage nature of carcinogenesis it is probable that large adenomas will have undergone several gene or chromosome mutations before reaching the fully malignant state.

In normal people loss or mutation of one copy of their two normal alleles of the APC gene would be expected to be an early event to initiate the requisite hyperproliferation for adenoma formation. Loss of chromosome 5 alleles would thus be expected in some small sporadic adenomas, of which we have one example. Chromosome instability would presumably be conferred by the heterozygous state, providing a mechanism for further genetic change by means of deletion or somatic crossing over leading to homozygosity or functional hemizygosity for critical loci on chromosome 5 or on chromosomes 17, 18 and 22. The latter chromosomes have recently been implicated in colorectal cancer by cytogenetic (Muleris *et al.*, 1987) and molecular data (Fearon *et al.*, 1987; Okamoto *et al.*, 1988). Use of polymorphic DNA probes which are closer to the critical region of chromosome 5 as well as those assigned to chromosomes 17, 18 and 22 will enable us to obtain a more complete picture of the genetic events leading from adenoma to carcinoma in both polyposis patients and normal individuals.

We wish to thank the following: all the staff of the Polyposis Registry, St Mark's Hospital, London, Mr A. Gunn, Ashington Hospital, Northumberland and Cmdr A.R. Mugridge, The Royal Naval Hospital, Plymouth for supplying material; Dr C. Paraskeva, The Medical School, Bristol for the cell line JW2, and Dr A. Jefferies and ICI Diagnostics for the minisatellite probes. M. Rees was supported by the Medical Research Council of the UK and S.E.A. Leigh by the Cancer Research Campaign.

### References

BODMER, W.F., BAILEY, C.J., BODMER, J. & 10 others (1987). Localization of the gene for familial adenomatous polyposis on chromosome 5. *Nature*, **238**, 614.

BULOW, S. (1987). Familial polyposis coli. *Dan. Med. Bull.*, **34**, 1.

BUSSEY, H.J.R. (1975). *Familial Polyposis Coli.* Johns Hopkins University Press: New York.

CAVANEE, W.K., DRYJA, T.P., PHILLIPS, R.A. & 6 others (1983). Expression of recessive alleles by chromosomal mechanisms in retinoblastoma. *Nature*, **305**, 779.

DELHANTY, J.D.A., DAVIS, M.B. & WOOD, J. (1983). Chromosome instability in lymphocytes, fibroblasts, and colon epithelial-like cells from patients with familial polyposis coli. *Cancer Genet. Cytogenet.*, **8**, 27.

FEARON, E.R., FEINBERG, A.P., HAMILTON, S.R. & VOGELSTEIN, B. (1985). Loss of genes on the short arm of chromosome 11 in bladder cancer. *Nature*, **318**, 377.

FEARON, E.R., HAMILTON, S.R. & VOGELSTEIN, B. (1987). Clonal analysis of human colorectal tumours. *Science*, **238**, 193.

FEINBERG, A.P. & VOGELSTEIN, B. (1983). A technique for radiolabelling DNA restriction endonuclease fragments to high specific activity. *Anal. Biochem.*, **132**, 6.

FEINBERG, S.M., JAGELMAN, D.G., SARRE, R.G. & 5 others (1988). Spontaneous resolution of rectal polyps in patients with familial polyposis following abdominal colectomy and ileorectal anastomosis. *Dis. Col. Rectum*, **31**, 169.

GARDNER, E.J., ROGERS, S.W. & WOODWARD, S. (1982). Numerical and structural chromosomal aberrations in cultured lymphocytes and cutaneous fibroblasts of patients with multiple adenomas of the colo-rectum. *Cancer*, **49**, 1413.

GRIFFITHS, D.F.R., DAVIES, S.J., WILLIAMS, D., WILLIAMS, G.T. & WILLIAMS, E.D. (1988). Demonstration of somatic mutation and colonic crypt clonality by x-linked enzyme histochemistry. *Nature*, **333**, 461.

HUMAN GENE MAPPING 9 (1987). *Cytogenet. Cell Genet.*, **46**, Nos. 1–4, 1.

KNUDSON, A.G. (1971). Mutation and cancer: a statistical study of retinoblastoma. *Proc. Natl Acad. Sci. USA*, **68**, 820.

KOK, K., OSINGA, J., CARRITT, B. & 9 others (1987). Deletion of a DNA sequence at the chromosomal region 3p21 in all major types of lung cancer. *Nature*, **330**, 578.

KOUFOS, A., HANSEN, M.F., LAMPKIN, B.C. & 4 others (1984). Loss of alleles at loci on human chromosome 11 during genesis of Wilms' tumour. *Nature*, **309**, 170.

KOUFOS, A., HANSEN, M.F., COPELAND, N.G., JENKINS, N.A., LAMPKIN, B.C. & CAVANEE, W.K. (1985). Loss of heterogeneity in three embryonal tumours suggests a common pathogenetic mechanism. *Nature*, **316**, 330.

LAW, O.J., OLSCHWANG, S., MONPEZAT, J.P. & 5 others (1988). Concerted nonsyntenic allelic loss in human colorectal carcinoma. *Science*, **241**, 961.

LEPPERT, M., DOBBS, M., SCAMBLER, P. & 11 others (1987). The gene for familial polyposis coli maps to the long arm of chromosome 5. *Science*, **238**, 1411.

LIPKIN, M. (1988). Biomarkers of increased susceptibility to gastrointestinal cancer: new application to studies of cancer prevention in human subjects. *Cancer Res.*, **48**, 235.

MANIATIS, T., FRITSCH, E.F. & SAMBROOK, J. (1982). *Molecular Cloning – A Laboratory Manual.* Cold Spring Harbor Laboratory: New York.

MULERIS, M., SALMON, R.-J., DUTRILLAUX, A.-M. & 4 others (1987). Characteristic chromosomal imbalances in 18 near-diploid colorectal tumours. *Cancer Gent. Cytogenet.*, **29**, 289.

MUTO, T., BUSSEY, H.J.R. & MORSON, B.C. (1977). The evolution of cancer of the colon and rectum. *Cancer*, **36**, 2251.

OKAMOTO, M., SASAKI, M., SUGIO, K. & 6 others (1988). Loss of constitutional heterozygosity in colon carcinoma from patients with familial polyposis coli. *Nature*, **331**, 273.

ORKIN, S.H., GOLDMAN, D.S. & SALLAN, S.E. (1984). Development of homozygosity for chromosome 11p markers in Wilms' tumour. *Nature*, **309**, 172.

PARASKEVA, C., BUCKLE, B.G., SHEER, D. & WIGLEY, C.B. (1984). The isolation and characterization of colorectal epithelial cell lines at different stages in malignant transformation from familial polyposis coli patients. *Int. J. Cancer*, **34**, 49.

REICHMANN, A., MARTIN, P. & LEVIN, B. (1984). Chromosomes in human large bowel tumours. A study of chromosome 1. *Cancer Genet. Cytogenet.*, **12**, 295.

REICHMANN, A., MARTIN, P. & LEVIN, B. (1985). Chromosomal banding patterns in human large bowel adenomas. *Human. Genet.*, **70**, 28.

SOLOMON, E., VOSS, R., HALL, V. & 6 others (1987). Chromosome 5 allele loss in human colorectal carcinomas. *Nature*, **328**, 616.

VAN DEN BERGHE, H. (1987). Second international workshop on chromosomes in solid tumours – conference summary. *Cancer Genet. Cytogenet.*, **28**, 49.

WONG, Z., WILSON, V., PATEL, I., POVEY, S. & JEFFREYS, A.J. (1987). Characterization of a panel of highly variable minisatellites cloned from human DNA. *Ann. Hum. Genet.*, **51**, 269.